# Synergistic antitumor effects of S-1 with eribulin *in vitro* and *in vivo* for triple-negative breast cancer cell lines

**DOI:** 10.1186/2193-1801-3-417

**Published:** 2014-08-08

**Authors:** Masato Terashima, Kazuko Sakai, Yosuke Togashi, Hidetoshi Hayashi, Marco A De Velasco, Junji Tsurutani, Kazuto Nishio

**Affiliations:** Department of Genome Biology, Kinki University Faculty of Medicine, 377-2 Ohno-higashi, Osaka-Sayama, Osaka, 589-8511 Japan; Medical Oncology, Kinki University Faculty of Medicine, 377-2 Ohno-higashi, Osaka-Sayama, Osaka, 589-8511 Japan

**Keywords:** TNBC, S-1, 5-FU, Eribulin, EMT, MET

## Abstract

Triple-negative breast cancer (TNBC) is associated with a higher incidence of recurrence and distant metastasis and a poor prognosis, whereas effective treatment strategies remain to be established. Finding an effective treatment for TNBC has become imperative. We examined the effect of the combination of S-1 (or 5-FU in an in vitro study) and eribulin in TNBC cell lines. The in vitro effect of the combination was examined in four TNBC cell lines (MDA-MB-231, MDA-MB-468, BT-549 and MX-1) using a combination index and isobolograms. In addition, we assessed the effect of the combination in an MDA-MB-231 tumor xenograft model. A synergistic effect was observed in three TNBC cell lines (MDA-MB-231, MDA-MB-468, and MX-1), and in an in vivo study, the combination of S-1 and eribulin resulted in significantly higher antitumor effects compared with S-1 or eribulin alone. 5-FU induced epithelial-mesenchymal transition (EMT) change in the TNCB cell line, as supported by the decreased expression of epithelial marker and the increased expression of mesenchymal markers. Meanwhile, TGF-beta induced EMT changes in a TNBC cell line and decreased the sensitivity to 5-FU. This result suggests that 5-FU-induced EMT changes reduce the sensitivity to 5-FU. In contrast, eribulin induced a mesenchymal-epithelial transition (MET) in a TNBC cell line. The EMT phenotype induced by 5-FU was also canceled by eribulin. We demonstrate that the combination of S-1 (5-FU) and eribulin exerts a synergistic effect for TNBC cell lines through MET-induction by eribulin. Therefore, this combination therapy may be a potential treatment option for TNBC.

## Introduction

Metastatic breast cancer is a heterogeneous disease in its biology, clinical behavior, prognosis, and treatment. Whereas patients with hormone receptor-positive and HER-2-positive breast cancers have had favorable outcomes with chemotherapy and targeted therapies, triple-negative breast cancer (TNBC) is not sensitive to conventional chemotherapies. TNBC is a clinical phenotype characterized by a lack of overexpression or gene amplification of hormone receptors (estrogen receptor [ER] and progesterone receptor [PR]) and human epidermal growth factor receptor-2 (*HER-2*). TNBC accounts for between 9% and 16% of all breast cancers and is associated with a poor prognosis (Foulkes et al. 2010; Blows et al. 2010; Curtis et al. 2012; Network 2012; Montagna et al. 2013).

S-1 (Taiho Pharmaceutical Co., Tokyo, Japan) is an oral fluoropyrimidine derivative composed of 1-(2-tetrahydrofuryl)-5-fluorouracil (tegafur, a prodrug of 5-flurouracil [5-FU]), combined with two modulators of 5-FU activity, 5-chloro-2, 4-dihydroxypyrimidine (gimeracil) and potassium oxonate (oteracil) (Shirasaka et al. 1996). S-1 has been widely approved for gastric cancer treatment in many countries in Asia and Europe (Satoh & Sakata 2012) and for several other cancers in Japan (Shirasaka 2009). In a phase II clinical study, S-1 monotherapy was demonstrated to have high antitumor activity with tolerated toxicities in metastatic breast cancer (Saek et al. 2004). Moreover, it was reported at the Annual Meeting of The Japanese Breast Cancer Society (Futsuhara 2012) that an additional treatment with S-1 for 12 months was acceptable for TNBC following treatment with anthracycline or taxane.

Eribulin mesylate (eribulin; Eisai Co., Tokyo, Japan) is a non-taxane, microtubule dynamics inhibitor with a novel mechanism of the action, which induces irreversible mitotic block at the G2-M phase and apoptosis (Kuznetsov et al. 2004; Towle et al. 2001; Jordan et al. 2005; Okouneva et al. 2008; Smith et al. 2010; Towle et al. 2011). In a randomized clinical phase III study (Eisai Metastatic Breast Cancer Study Assessing Physician’s Choice Versus E7389, EMBRACE) involving patients with heavily pre-treated locally recurrent or metastatic breast cancer, eribulin was compared with the treatment of physician’s choice (TPC). Patients who received eribulin exhibited a significant improvement in median overall survival compared with TPC (Cortes et al. 2011). Recently, sub-group analysis from phase III clinical trial demonstrated that women with TNBC had significant response to treatment with eribulin versus capecitabine, with a median overall survival of 14.4 months with eribulin, compared with 9.4 months with capecitabine (Kaufman et al. 2012).

Because a monotherapy of S-1 or eribulin has shown promising activity in TNBC and eribulin have a manageable toxicity and a modest incidence of neuropathy, which appears to be lower than with other microtubule inhibitor agents, the combination of S-1 and eribulin is a hopeful combination regimen for breast cancer including TNBC. Phase I trial of S-1 and eribulin combination therapy for advanced or recurrent breast cancer pretreated by anthracycline and taxane is being executed in Kinki University Hospital. However, no preclinical studies of this combination have been performed. In this study, we investigated the combination effect of S-1 (5-FU) and eribulin on TNBC cell lines both *in vitro* and *in vivo*. In addition, we investigated the mechanisms of the synergistic effects of S-1 (5-FU) and eribulin.

## Methods

### Cell lines

This study was performed using four TNBC cell lines (MDA-MB-231, MDA-MB-468, BT-549, and MX-1) and two non-TNBC cell lines (MCF-7, T47D). These cells were obtained from ATCC (Rockville, MD, USA) or CLS (Eppelheim, Germany) and were maintained in RPMI-1640 medium (Sigma-Aldrich, St. Louis, MO, USA) supplemented with 10% fetal bovine serum (Gibco BRL, Grand Island, NY, USA) and cultured under a humidified atmosphere of 5% CO_2_ at 37°C, passaged every 3–4 days.

### Compounds

S-1 was prepared by mixing tegafur, gimeracil, and oteracil potassium (TCI, Tokyo, Japan) at a molar ratio of 1:0.4:1 in 0.5% HPMC. 5-FU was obtained from Sigma-Aldrich for the *in vitro* study and eribulin was provided by Eisai Co. These agents were dissolved in dimethyl sulfoxide (DMSO).

### In vitro growth inhibition assay

Cell growth was assessed using a standard 3-(4, 5-dimenthyl-thiazoyl-2-yl) 2, 5-diphenyltetrazolium bromide (MTT) assay and a previously described method (Tanaka et al. 2009). Briefly, cells were seeded into a 96-well plate and were cultured for 24 h before exposure to the compounds. The cells were incubated for 72 h at 37°C with various concentrations of 5-FU and eribulin. The experiment was performed in triplicate.

### Combination effect of 5-FU and eribulin in vitro

To evaluate the combination effect, the normalized ED_50_ isobologram was plotted using an MTT assay. An isobologram analysis is a frequently used method for analyzing the effects of multiple drugs (Steel & Peckham 1979; Kano et al. 1988). In addition, the combination index (CI) (Chou & Talalay 1984) was calculated for each combination ratio using the following formula:


where (Da)_1_ and (Da)_2_ are the concentrations required for single agents to achieve a% drug effect (in this experiment, a = 50) and (D)_1_ and (D)_2_ are the concentrations of 5-FU and eribulin used in combination to achieve the same effect. In a normalized isobologram, the diagonal line represents the additive effect. Experimental data points, represented by dots located below, on, or above the line, indicate synergism, additively, and antagonism, respectively. The CI equation determines the additive effect of drug combinations, with synergism being defined as greater than the expected additive effect and antagonism being defined as less than the expected additive effect. Thus, CI values <1, 1, and >1 indicate synergism, additively and antagonism, respectively.

### Real-time reverse-transcription (RT) PCR

Total RNA was converted to cDNA using a GeneAmp® RNA-PCR kit (Applied Biosystems, CA, USA). The cDNAs were used for PCR analysis using oligonucleotide primers specific for E-cadherin (forward 5’-TTAAACTCCTGGCCTCAAGCAATC-3’ and reverse 5’-TCCTATCTTGGGCAAAGCAACTG-3’) N-cadherin (forward 5’-CGAATGGATGAAAAGACCCATCC-3’ and reverse 5’-GGAGCCACTGCCTTCATCGTCAA-3’) vimentin (forward 5’-TGAGTACCGGAGACAGGTGCAG-3’ and reverse 5’-TAGCAGCTTCAACGGCAAAGTTC-3’) and Snail2 (forward 5’-ATGCATATTCGGACCCACACATTAC-3’ and reverse 5’-AGATTTGACCTGTCTGCAAATGCTC-3’). The PCR was performed using Thermal Cycler Dice (TaKaRa, Otsu, Japan) under the following conditions: 95°C for 5 min, 50 cycles of 95°C for 5 s, and 60°C for 10 s. The primers were purchased from Sigma-Aldrich or TaKaRa and were used with SYBR® Premix Ex Taq (TaKaRa). GAPDH was used as an internal control to normalize and compare each sample.

### Immunoblotting

Immunoblot analysis was performed as described previously (Maegawa et al. 2009). Cells were washed twice with phosphate-buffered saline (PBS) and lysed by incubating in Lysis A buffer containing 1% Triton X-100, 20 mM Tris–HCl (pH7.0), 5 mM EDTA, 50 mM sodium chloride, 10 mM sodiumpyrophosphate, 50 mM sodium fluoride, 1 mM sodium orthovanadate, a protease inhibitor cocktail tablet (Complete, Mini; Roche Diagnostics, Basel, Switzerland) and phosphatase inhibitor cocktail (Sigma-Aldrich). Proteins were resolved using SDS-PAGE and were transferred to a PVDF membrane (Immobilon; Millipore, Billerica, MA, USA). After blocking with Tris-buffered saline (TBS) containing 0.02% Tween 20 and 5% nonfat milk, the strips of membrane were exposed to anti-E-cadherin antibody, anti-N-cadherin antibody, anti-vimentin antibody, anti-Snail2 antibody, or anti-β-actin antibody. They were incubated with HRP-conjugated anti-rabbit IgG antibody and the proteins were visualized using an ECL Western Blotting Detection System (GE Healthcare, Buckinghamshire, UK). The antibodies were all purchased from Cell Signaling (Beverly, MA, USA).

### Immunofluorescence staining

Immunofluorescence staining of cells were performed as described previously (Tamura et al. 2010) with minor modifications. Cells were seeded onto cover slips in 6-well plates and exposed to 5-FU, eribulin, or TGF-β. After exposure, the cells were washed twice with PBS, then fixed with 4% paraformaldehyde for 20 min. The fixed cells were washed three times with PBS and were subsequently incubated for 1 hour with 1.5% bovine serum albumin. They were then incubated over night with anti-E-cadherin or anti-vimentin antibodies at 4°C, followed by incubation with Alexa Fluor 546 anti-rabbit IgG antibody (Molecular Probes, Eugene, OR, USA) for 30 min. Finally, the cells were treated with DAPI (6-diamidino-2-phenylindole) to stain the nucleus and the stained cells were observed using fluorescence microscopy (IX71; Olympus, Tokyo, Japan).

### In vivo growth inhibition assay

Female BALB/c-nu/nu mice (5 weeks old; CLEA Japan Inc., Tokyo, Japan) were used for the *in vivo* studies and were cared for in accordance with the recommendations for the handling of laboratory animals for biomedical research compiled by the Committee on Safety and Ethical Handling Regulations for Laboratory Animal Experiments, Kinki University. MDA-MB-231 cells (4 × 10^6^ viable cells) in PBS containing 50% Matrigel were injected subcutaneously into each mouse. Thirty days following the inoculation of the cells, the mice were divided randomly into 4 groups (control, S-1, eribulin, and S-1 plus eribulin; n = 3 each), and treatment was initiated. S-1 was administered orally on days 1 to 16, and eribulin dissolved in saline containing 2.5% DMSO was injected intravenously on days 1, 5, 9, and 13. The doses of S-1 and eribulin were 8.3 mg/kg and 0.1 mg/kg, respectively. The tumor volumes were estimated using the formula [(width)^2^ × length]/2 (mm^3^). To evaluate the anti-tumor effects of the S-1, eribulin, and a combination of S-1 and eribulin, the tumor sizes and body weights were measured twice per week. Antitumor effects were expressed as % T/C (treated versus control), dividing the tumor volumes from the treatment groups by those of the control groups and multiplying by 100.

### Hematoxylin-Eosin (HE) staining and immunohistochemical (IHC) analysis

Formalin-fixed tumors were paraffin-embedded. 4 μm sections were used for HE staining, and IHC using antibodies against E-cadherin (1:100) and to vimentin (1:100). The methods used in this section have been described previously (Tamura et al. 2013).

### Statistical analysis

The statistical analyses were performed using Microsoft Excel (Microsoft) to calculate the SD and to test for statistically significant differences between the samples using a Student's *t* test. A *P* value of <0.05 was considered statistically significant.

## Results

### Cellular sensitivity of TNBC cell lines to 5-FU and eribulin

The growth inhibitory effects of 5-FU and eribulin on TNBC cell lines and non-TNBC cell lines are examined using an MTT assay. The IC_50_ values of 5-FU and eribulin for the cell lines fell within the ranges of 2.3-13.0 μM and 0.4-4.3 nM, respectively. There was no difference in cellular sensitivity between the TNBC cell lines and the non-TNBC cell line, except for the MX-1 cells (Table 1). The MX-1 cells had a higher resistance to 5-FU (92.4 ± 3.7 μM) than the other cell lines.

**Table 1:** **IC50 value of 5-FU and eribulin**

Cell lines		5-FU (μM)	Eribulin (nM)
TNBC	MDA-MB-231	13.0 ± 2.0	1.2 ± 0.3
	MDA-MB-468	8.6 ± 2.5	0.7 ± 0.2
	BT-549	5.3 ± 0.5	1.6 ± 0.2
	MX-1	92.4 ± 3.7	4.3 ± 0.6
Non-TNBC	MCF-7	2.3 ± 0.4	0.4 ± 0.0
	T47D	5.1 ± 0.4	2.6 ± 1.1

Growth inhibitory effect of single agent 5-FU or eribulin in four TNBC cell lines and two non-TNBC cell lines. MTT assays were performed. The IC_50_ values of each agent were shown at 72 hours after exposure. Each value is the average ± SD of three independent experiments.

### In vitro combination effect of 5-FU and eribulin on TNBC cell lines

**Figure 1:**
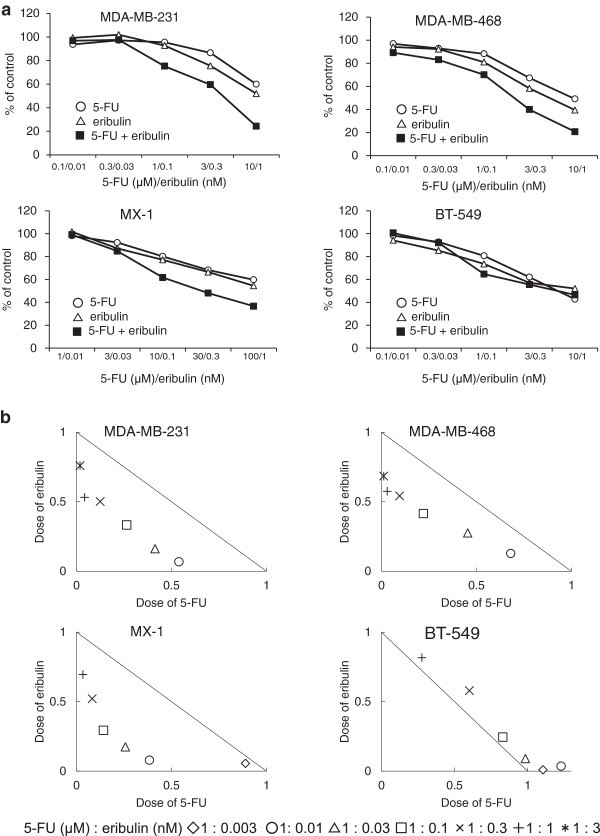
**Combination effect of 5-FU and eribulin in four TNBC cell lines. (a)** Growth inhibitory effect of combination of 5-FU and eribulin in TNBC cell lines after 72 hours exposure. **(b)** Normalized isobologram analysis. The diagonal line represents the additive effect. Experimental data points, represented by dots located below, on, or above this line indicate synergism, additively, and antagonism, respectively. Points located below and to the left of the line represent synergism.

To evaluate the potential combined effect of 5-FU and eribulin, normalized ED_50_ isobolograms were plotted and the CI values were determined using an MTT assay. A synergistic interaction between 5-FU and eribulin was observed in the TNBC cell lines (MDA-MB-231, MDA-MB-468, and MX-1) (Figure 1a). In addition, the CI values were significantly <1 and isobolograms clearly revealed synergy at all explored concentrations (Table 2 and Figure 1b). An additive effect was observed in the BT-549 cell line. Thus, we found a synergistic or additive effect for the combination of 5-FU and eribulin in the TNBC cell lines.

**Table 2:** **CI value of combination of 5-FU and eribulin**

Combination ratio	Cell lines			
5-FU (μM): eribulin (nM)	MDA-MB-231	MDA-MB-468	MX-1	BT-549
1 : 0.003			0.95	1.12
1 : 0.01	0.61	0.81	0.46	1.27
1 : 0.03	0.58	0.73	0.27	1.08
1 : 0.1	0.60	0.63	0.44	1.08
1 : 0.3	0.63	0.64	0.60	1.18
1 : 1	0.57	0.61	0.73	1.09
1 : 3	0.61	0.70		

The CI values were calculated in each combination ratio. The CI values of <1, 1, or >1 indicate synergism, additively, and antagonism, respectively.

### EMT changes reduce the sensitivity of MDA-MB-231 cells to 5-FU

**Figure 2:**
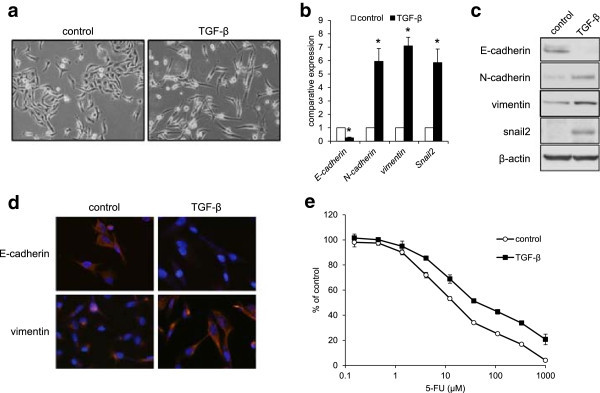
**TGF-β induces EMT in MDA-MB-231 cells after 5 days of exposure and reduced the sensitivity to 5-FU. (a)** Morphological changes of MDA-MB-231 cells after exposure to 2.5 ng/mL of TGF-β. **(b)** The mRNA expression levels of E-cadherin, N-cadherin, vimentin and Snail2 were determined using real-time RT-PCR. GAPDH was used to normalize the expression levels. The data shown represent the average ± SD of three independent experiments. *P < 0.05 versus control. **(c)** Western blotting analysis for E-cadherin, N-cadherin, vimentin, and Snail2. β-actin was used as an internal control. **(d)** Immunofluorescence staining of E-cadherin and vimentin. The nucleus (blue) was stained with DAPI. **(e)** Cellular growth inhibition curves of 5-FU in MDA-MB-231 cells with or without TGF-β exposure.

We analyzed the mechanisms of the synergistic interaction between 5-FU and eribulin. Several reports have demonstrated that established 5-FU-resistant cell lines exhibit EMT changes (Chung et al. 2000; Zhang et al. 2012), we examined focusing on EMT. First, we examined whether EMT changes in MDA-MB-231 cells might reduce the cellular sensitivity to 5-FU. When MDA-MB-231 cells were exposed to TGF-β (2.5 ng/mL) for 5 days, an EMT-like morphological change was observed (Figure 2a). The decreased expression of E-cadherin and the increased expression of N-cadherin, vimentin and Snail2 were observed at the mRNA and protein expression levels (Figure 2b and c). Immunofluorescence staining confirmed these changes in E-cadherin and vimentin (Figure 2d). Figure 2e shows the cellular sensitivity of MDA-MB-231 cells to 5-FU under this condition. The cells that were exposed to TGF-β had an approximately 3-fold higher resistance to 5-FU, with IC_50_ values of 44.7 ± 5.8 μM, compared with that of cells without TGF-β exposure (14.8 ± 1.0 μM). These results showed the EMT changes in MDA-MB-231 cells reduced cellular sensitivity to 5-FU.

### 5-FU induces epithelial-mesenchymal transition (EMT)

**Figure 3:**
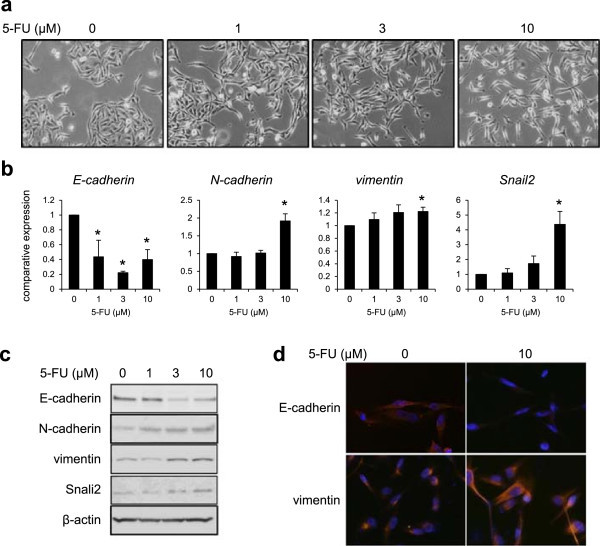
**5-FU induces EMT in MDA-MB-231 cells after 5 days of exposure. (a)** Morphological changes in MDA-MB-231 cells after exposure to 1, 3, or 10 μM of 5-FU. **(b)** The mRNA expression levels of E-cadherin, N-cadherin, vimentin and Snail2 were determined using real-time RT-PCR. GAPDH was used to normalize the expression levels. The data shown represent the average ± SD of three independent experiments. *P < 0.05 versus control. **(c)** Western blotting analysis for E-cadherin, N-cadherin, vimentin, and Snail2. β-actin was used as an internal control. **(d)** Immunofluorescence staining of E-cadherin and vimentin. The nucleus (blue) was stained with DAPI.

When MDA-MB-231 cells were treated with 5-FU (1–10 μM), we observed that MDA-MB-231 cells displayed EMT-like changes in cell morphology, including the elongation of the cell shape and cell scattering, which are characteristic features of cells undergoing EMT (Figure 3a). Real-time RT-PCR and western blotting demonstrated the decreased of an epithelial marker (E-cadherin), whereas the expressions of mesenchymal markers (N-cadherin and vimentin) and Snail2, which are transcriptional repressors of E-cadherin, were increased after 5 days of 5-FU exposure in dose-dependent manners (Figure 3b and c). The decreased expression of E-cadherin and the increased expression of vimentin were also confirmed using immunofluorescence staining (Figure 3d). These results indicated that 5-FU directly induced EMT in MDA-MB-231 cells and the EMT changes might reduce the sensitivity to 5-FU.

### Eribulin induces mesenchymal-epithelial transition (MET)

**Figure 4:**
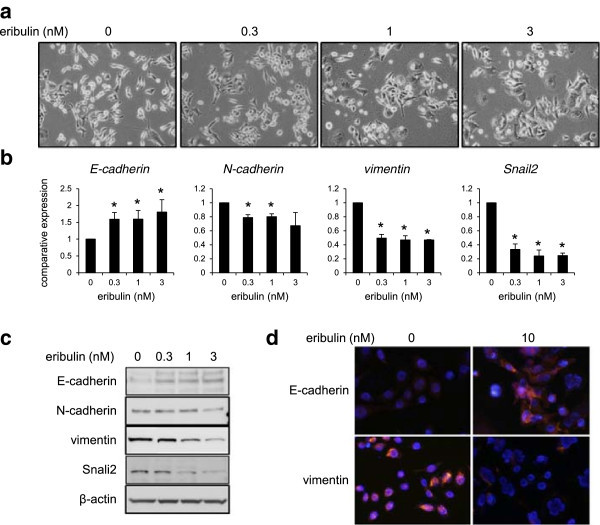
**Eribulin induces MET in MX-1 cells after 8 days of exposure. (a)** Morphological changes of MX-1 cells after exposure to 1, 3, or 10 nM of eribulin. **(b)** The mRNA expression levels of E-cadherin, N-cadherin, vimentin and Snail2 were determined using real time RT-PCR. GAPDH was used to normalize the expression levels. The data shown represent the average ± SD of three independent experiments. *P < 0.05 versus control. **(c)** Western blotting analysis for E-cadherin, N-cadherin, vimentin, and Snail2. β-actin was used as an internal control. **(d)** Immunofluorescence staining of E-cadherin (red) and vimentin (red). The nucleus (blue) was stained with DAPI.

**Figure 5:**
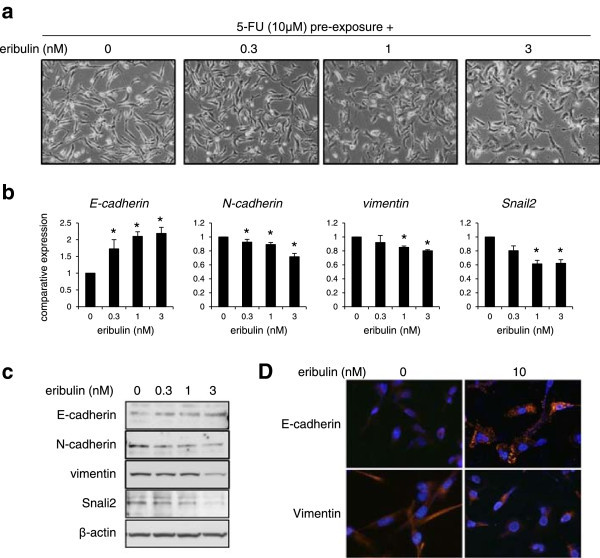
**Eribulin induces MET in MDA-MB-231 cells in which EMT changes have been induced by 5-FU. (a)** Morphological changes of MDA-MB-231 cells after 5 days of 5-FU treatment and 3 additional days of eribulin treatment. **(b)** The mRNA expression levels of E-cadherin, N-cadherin, vimentin and Snail2 were determined using real time RT-PCR. GAPDH was used to normalize the expression levels. The data shown represent the average ± SD of three independent experiments. *P < 0.05 versus control. **(c)** Western blotting analysis for E-cadherin, N-cadherin, vimentin, and Snail2. β-actin was used as an internal control. **(d)** Immunofluorescence staining of E-cadherin (red) and vimentin (red). The nucleus (blue) was stained with DAPI.

In contrast, MX-1 cells, which exhibit mesenchymal features, displayed MET morphologically changes including a cobblestone-like appearance and tight cell–cell junctions, the reverse process of EMT, after 8 days exposure to eribulin (0.3-3 nM) (Figure 4a). In agreement with these observations, the increased expression of E-cadherin and the decreased expression of N-cadherin and vimentin in association with decreased expression of Snail2 were detected in dose-dependent manners (Figure 4b and c). Immunofluorescence staining also confirmed the increased expression of E-cadherin and the decreased expression of vimentin (Figure 4d). To examine whether eribulin cancels 5-FU-induced EMT changes in MDA-MB-231 cells, the cells were pre-exposed to 5-FU for 5 days, followed by exposure to eribulin. Morphological changes from fibroblast-like shapes to a cobblestone appearance were observed after eribulin-exposure (0.3-3 nM) for 4 days (Figure 5a). The increased expression of E-cadherin and the decreased expression of N-cadherin, vimentin and Snail2 were also observed in dose-dependent manners, as detected using real-time RT-PCR, western blotting, and immunofluorescence staining (Figures 5b-d). Thus, the 5-FU induced EMT phenotype in MDA-MB-231 cells was also canceled by eribulin. These results suggested the action of MET induction by eribulin reduced the 5-FU resistance.

### Combination effect of S-1 and eribulin in an MDA-MB-231 tumor xenograft model

**Figure 6:**
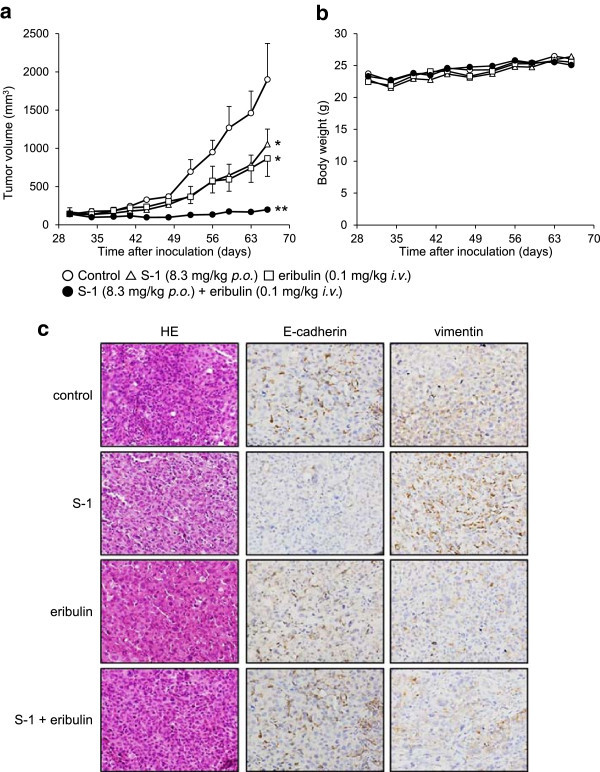
**Combination effects of S-1 and eribulin in an** ***in vivo*** **xenograft model. (a)** The tumor volumes of the four groups are plotted from day 30 until day 66 after the inoculation of MDA-MB-231 cells. The data shown represent the average values (bars, SD). *P < 0.05 versus control, **P < 0.05 versus S-1 or eribulin alone. **(b)** Body weight loss in mice. **(c)** HE staining assay and IHC assays using mouse tumor samples. Tumors were collected after 7 days of treatment and the prepared tissue slides were analyzed with HE staining and IHC using anti-E-cadherin and anti-vimentin antibodies.

To assess the combination effect of S-1 and eribulin *in vivo*, we examined the antitumor activity of this combination in an MDA-MB-231 tumor xenograft model. Mice bearing MDA-MB-231 tumors were prepared and treated with S-1 (days 1–16; p.o., 8.3 mg/kg), eribulin (Q4D × 4; i.v., 0.1 mg/kg), or S-1 plus eribulin. The mean tumor volumes (mm^3^) on day 36 following the initial treatment of the 4 groups (*i.e.*, control, S-1, eribulin, and S-1 plus eribulin) were 1900 ± 473, 1055 ± 197, 900 ± 235, and 199 ± 26, respectively (Figure 6a, *P* = 0.01, S-1 monotherapy versus S-1 plus eribulin; *P* = 0.04, eribulin versus S-1 plus eribulin). S-1 or eribulin alone inhibited the tumor growth (T/C = 55.5%, 47.4%, respectively), and a combination of S-1 and eribulin inhibited the tumor growth of MDA-MB-231 xenografts more intensively (T/C = 10.5%). Body weight loss related to treatment with S-1 or eribulin was not observed in any of the groups (Figure 6b). Further, we examined the MET induction activity of eribulin using a xenograft model. Immunohistchemical analyses were performed in above four groups on day 7 following the initial treatment. As a result, the decreased expression of E-cadherin and the increased expression of vimentin were observed after treatment of S-1. In contrast, the increased expression of E-cadherin and the decreased expression of vimentin were observed after treatment of combined with eribulin (Figure 6c). These results were consistent with the *in vitro* experiments.

## Discussion

Patients with advanced or metastatic breast cancer have limited treatment options. Patients with TNBC can receive systemic therapy only due to lack of targeted therapies and many patients fail to respond or become refractory to the treatment. In spite of high response rate to chemotherapy in the initial treatment, TNBC patients develop rapid disease progression resulting in a shorter overall survival compared to ER-positive breast cancer (Foulkes et al. 2010). Metastatic breast cancer is an incurable disease and the purpose of chemotherapy is relieving symptoms and improving quality of life. Although combination chemotherapies such as docetaxel-capecitabine were demonstrated superior efficacy for metastatic breast cancer, nonhematologic toxicities were significantly higher (O'Shaughnessy et al. 2002). Recently, in studies with selected patients referred for BRCA genetic testing the frequency of TNBC has been reported to be 57% in BRCA1 mutation carriers, and 23% in BRCA2 mutation carriers (Atchley et al. 2008). Several PARP inhibitors are being tested in clinical trials such as olaparib, which has been shown to be safe and effective in BRCA-related cancers. However, the benefit of iniparib in phase II trial was not confirmed in the subsequent phase III trial (Audeh et al. 2010). Thus, novel therapies and treatment modalities for TNBC are necessary.

In this study, we demonstrated that the combination of 5-FU and eribulin exerted synergistic (3/4) or additive (1/4) effects against TNBC cell lines *in vitro*. The synergistic effect of S-1 with eribulin was also demonstrated in a tumor-bearing model. In the *in vivo* experiment, we analyzed the antitumor effects of S-1 and eribulin according to the optimal dose and schedule for each drug. The combination was tolerable and resulted in the remarkable reduction of tumor growth in mice without remarkable toxicities including body-weight loss and diarrhea. These preclinical studies are first reports and these results show that the combination may be potential therapy for TNBC patients.

EMT has emerged to play important roles in the development of the invasive and metastatic potentials of cancer progression (Hugo et al. 2007; Peinado et al. 2007; Tsuji et al. 2009). In addition to this action, recent evidence indicates that EMT changes develop a resistance to a several anti-cancer agents such as EGFR-tyrosine kinase inhibitor, cisplatin, gemcitabine, and 5-FU (Singh & Settleman 2010; Thomson et al. 2005; Frederick et al. 2007; Zhuo et al. 2008; Arumugam et al. 2009; Wang et al. 2009). Actually, MX-1 cells, which assume intense mesenchymal feature, show low sensitivity to 5-FU and we ascertained that TGF-β-induced EMT change in TNBC cell line showed remarkably resistant to 5-FU. Furthermore, 5-FU directly induced EMT change in TNBC cells, and this action of 5-FU is likely to be associated with acquired resistance. In contrast, the present study has shown that eribulin induced MET, and current preclinical studies represent that eribulin reverses EMT and induce MET in TNBC cells through regulating TGF-β signal pathway, especially down-regulation of Smad2 and Smad3 phosphorylation (Yoshida et al. 2014). Our results were consistent with the report and eribulin moreover reversed the 5-FU-induced EMT in TNBC cells. These results were conformed both of *in vitro* and *in vivo* experiments. The action of eribulin on MET may improve the 5-FU resistance, resulting in produced a synergistic effect. Because of evidence of an association between EMT change and resistance to several other anti-cancer agents, these actions of eribulin provide a rationale for the combination of these agents. Moreover, a previous report demonstrated that a portion of TNBC tumors among clinical samples exhibit EMT changes and these subsets are significantly associated with a high histological grade (Jeong et al. 2012). Therefore, our combination treatment is expected to be a useful treatment strategy for EMT-positive TNBC.

In conclusion, we showed that the combination of S-1 (5-FU) and eribulin exerts a synergistic anti-tumor effect against TNBC cell lines *in vitro* and *in vivo* through the MET induction by eribulin. This combination may be beneficial to TNBC treatment, and the present evidence provides a good rationale to clinical studies for metastatic breast cancer patients. Currently, we are conducting a clinical study of S-1 and eribulin combination for metastatic breast cancer and clinical benefit of this regimen is being evaluated.

## Abbreviations

CI: Combination index
EMT: Epithelial-mesenchymal transition
ER: Estrogen receptor
eribulin: Eribulin mesylate
HE: Hematoxylin-eosin
HER-2: Human epidermal growth factor receptor-2
IHC: Immunohistochemical
MET: Mesenchymal-epithelial transition
PR: Progesterone receptor
RT: Reverse-transcription
SD: Standard deviation
TNBC: Triple-negative breast cancer
5-FU: 5-Fluorouracil.
